# Discovery and comparative genomic analysis of a novel equine anellovirus, representing the first complete *Mutorquevirus* genome

**DOI:** 10.1038/s41598-023-30875-7

**Published:** 2023-03-06

**Authors:** Mathew Fisher, Michelle Nebroski, Jennifer Davies, Eugene Janzen, Daniel Sullivan, Oliver Lung

**Affiliations:** 1 National Centre for Foreign Animal Disease, Canadian Food Inspection Agency, Winnipeg, MB Canada; 2 Faculty of Veterinary Medicine, University of Calgary, Calgary, AB Canada; 3 Department of Biological Sciences, University of Manitoba, Winnipeg, MB Canada

**Keywords:** Bioinformatics, Virology

## Abstract

The complete genome of a novel torque teno virus species (*Torque teno equus virus 2* (TTEqV2) isolate Alberta/2018) was obtained by high-throughput sequencing (HTS) of nucleic acid extracted from the lung and liver tissue of a Quarter Horse gelding that died of nonsuppurative encephalitis in Alberta, Canada. The 2805 nucleotide circular genome is the first complete genome from the *Mutorquevirus* genus and has been approved as a new species by the International Committee on Taxonomy of Viruses. The genome contains several characteristic features of torque teno virus (TTV) genomes, including an ORF1 encoding a putative 631 aa capsid protein with an arginine-rich N-terminus, several rolling circle replication associated amino acid motifs, and a downstream polyadenylation signal. A smaller overlapping ORF2 encodes a protein with an amino acid motif (WX_7_HX_3_CXCX_5_H) which, in general, is highly conserved in TTVs and anelloviruses. The UTR contains two GC-rich tracts, two highly conserved 15 nucleotide sequences, and what appears to be an atypical TATA-box sequence also observed in two other TTV genera. Codon usage analysis of TTEqV2 and 11 other selected anelloviruses from five host species revealed a bias toward adenine ending (A3) codons in the anelloviruses, while in contrast, A3 codons were observed at a low frequency in horse and the four other associated host species examined. Phylogenetic analysis of TTV ORF1 sequences available to date shows TTEqV2 clusters with the only other currently reported member of the *Mutorquevirus* genus, Torque teno equus virus 1 (TTEqV1, KR902501). Genome-wide pairwise alignment of TTEqV2 and TTEqV1 shows the absence of several highly conserved TTV features within the UTR of TTEqV1, suggesting it is incomplete and TTEqV2 is the first complete genome within the genus *Mutorquevirus*.

## Introduction

Torque teno viruses (TTVs) (family *Anelloviridae)* are non-enveloped viruses with small, circular, negative-sense, single-stranded DNA genomes that vary in length from 2.1 to 3.9 kb^1^. Anelloviruses are prevalent globally and have been reported in humans as well as a wide variety of wild and domestic animals, including non-human primates (e.g., chimpanzees, macaques, tamarin monkeys, and douroucouli), wild boars, badgers, pine martens, tupaias, rodents, bats, sea turtles, sea lions, livestock (e.g., pigs, sheep, cattle, camels, and poultry) and companion animals (e.g., cats and dogs)^1^. Several diseases have been proposed to be linked with TTV infection; however, there are few reports that support its potential as an etiological agent^1^. The International Committee on Taxonomy of Viruses (ICTV) currently recognizes 30 genera within *Anelloviridae.* Taxonomic classification of anelloviruses is based on nucleotide sequence similarity of ORF1 with cut-off values of 44% and 65%, respectively, for genus and species^2^.

TTV genomes reported to date consist of an untranslated region (UTR), two main open reading frames (ORFs), and may also have a variable number of additional ORFs. The UTR contains several conserved genomic features, including at least one GC-rich tract^3^ and several transcription elements^4,5^. Additionally, there are two 15 nucleotide conserved sequences (CGAATGGCTGAGTTT and AGGGGCAATTCGGGC) in the UTR of TTVs from both human and animal hosts^5–8^. ORF1 encodes a product of approximately 700–770 amino acids, which is considered the viral capsid protein^7^. Conserved amino acid motifs associated with rolling circle replication (Motif I: Fu[t/u][l/y][t/p], Motif II: [p/u]HuH and Motif III: YxxK) and helicase activity (Walker-A: GxxxxGK[S/T], Walker-B: hhxh[D/E][D/E] and motif C: h[T/S/x][T/S/x]N) observed in other single-stranded circular DNA viruses are also present in the ORF1 of some TTVs; however, there is no consistent trend in their presence^9^. ORF2 codes for a product of about 200 amino acids which contains a protein-tyrosine phosphatase amino acid motif (WX_7_HX_3_CXCX_5_H) found in both TTVs and chicken anemia virus (a gyrovirus)^10,11^ and is thought to be involved in cellular and/or viral protein regulation and processing during natural infection^5,12^.

Currently, there is only one publicly available sequence within the genus *Mutorquevirus*, Torque teno equus virus 1 (TTEqV1, KR902501); however, it is missing several features highly conserved within the UTR of TTV genomes. These missing features include one of the two highly conserved 15 nt sequences and a GC-rich region, suggesting that the UTR of the sequence currently available for TTEqV1 is incomplete. Here, we report the complete genome sequence of a novel TTV species, Torque teno equus virus 2 (TTEqV2) isolate Alberta/2018, discovered via high-throughput sequencing (HTS) of tissue samples collected from a horse that died of nonsuppurative encephalitis. The novel virus described here has been officially approved by the ICTV as a novel species within the genus *Mutorquevirus*^13^. The name of the novel virus complies with current ICTV naming guidelines, however switching to a binomial naming system has been suggested by the ICTV but not yet formally adopted^14^. When this system is formally adopted, the name of the novel virus would be changed to Mutorquevirus equid 2.

## Materials and methods

### Case history

The carcass of a 2-year-old Quarter Horse gelding from southern Alberta, Canada that died suddenly was submitted to the Diagnostic Services Unit at University of Calgary’s Faculty of Veterinary Medicine for post-mortem examination. Gross examination revealed lesions of trauma consistent with the horse being down and thrashing prior to death. Histopathology revealed severe nonsuppurative meningoencephalitis as the cause of death. Immunohistochemistry was negative for rabies virus, West Nile virus and *Sarcocystis neurona*. PCR was negative for eastern equine encephalitis virus and western equine encephalitis virus. Post-mortem liver, lung, spleen, brain and kidney tissue samples were submitted to the Canadian Food Inspection Agency’s National Center for Foreign Animal Disease Genomics Unit for characterization via HTS.

### Sample processing and high-throughput metagenomic sequencing

Tissue processing and HTS were performed as previously described^15–17^. Briefly, ten percent suspensions from the liver, lung, spleen, and kidney tissues were processed on a Precellys 24 Dual Tissue Homogenizer (Bertin Instruments). Nucleic acid extraction was performed using the Ambion MagMax Viral RNA Isolation Kit (Thermo Fisher Scientific) according to the manufacturer’s instructions and eluted in UltraPure water (Sigma-Aldrich). Brain tissue in formalin solution was processed separately using the Agencourt FormaPure Total kit (Beckman Coulter), designed to extract total nucleic acid from FFPE tissue. Since the brain tissue was not paraffin embedded, the “deparaffinization” step was omitted, but the manufacturer’s instructions were followed otherwise. Two extractions were prepared: one from the outside of the brain exposed directly to formalin and the second from the inside of the brain after cutting it in half.

To enable broad metagenomic detection of viruses with either DNA or RNA genomes, reverse transcription was performed separately on extracted nucleic acid from each tissue using the Invitrogen SuperScript IV First-Strand Synthesis System (SSIV) (Thermo Fisher Scientific) according to the manufacturer’s instructions using a tagged random nonamer primer (40 µM, GTT TCC CAG TCA CGA TAN NNN NNN NN). Sequenase Version 2.0 DNA Polymerase (Thermo Fisher Scientific) was used to perform second strand synthesis. Sequence-independent single-primer amplification (SISPA) was performed using AccuPrime Taq DNA Polymerase System (Thermo Fisher Scientific) with the manufacturer’s recommended conditions. Here, cDNA was amplified using a primer complementary to the tag introduced during reverse transcription. The SISPA product was purified using Genomic DNA Clean & Concentrator-10 columns (Zymo Research), quantified with the Qubit™ dsDNA HS Assay Kit on the Qubit® 3.0 Fluorometer (Thermo Fisher Scientific), and subsequently used for HTS library preparation.

Sequencing library preparation and enrichment were performed individually on each of the tissue derived samples using the Kappa HyperPlus library preparation kit (Roche Diagnostics) and a custom pan-vertebrate virus-targeted enrichment probe panel as previously described^16,18,19^. Following enrichment, pooled libraries were quantified for concentration (Qubit™ dsDNA HS Assay Kit on the Qubit® 3.0 Fluorometer (Thermo Fisher Scientific)) and fragment size (High Sensitivity DNA Kit on the 2100 Bioanalyzer (Agilent Technologies)) and sequenced on the Illumina MiSeq with a V3 flow cell using a 600 cycle kit (Illumina).

### Metagenomic sequencing assembly

Initial exploratory metagenomic analysis was done as previously described^16^. Briefly, an in-house developed automated taxonomic classification workflow (nf-villumina v2.0.0^20^) was used to analyze metagenomic sequencing data. First, nf-villumina removed Illumina PhiX Sequencing Control V3 reads using BBDuk^21^, followed by adaptor removal and quality filtering with fastp^22^. Filtered reads were taxonomically classified with Centrifuge^23^ and Kraken2^24^ using an NCBI nt Centrifuge index built February 14, 2020 and a Kraken2 index of NCBI RefSeq sequences of archaea, bacteria, viral and the human genome GRCh38 downloaded and built on March 22, 2019. Viral and unclassified reads were retained for de novo assembly with Unicycler^25^, Shovill^26^, and MEGAHIT^27^, and the resulting contigs from each were queried against the NCBI nr/nt database (downloaded January 9, 2020) using blastn (v2.9.0)^28^ (default parameters except “e-value 1e−6”). Contigs of interest were further analyzed in Geneious (v9.1.8)^29^ using a combination of reference assembly with unfiltered reads (default medium–low sensitivity settings and five iterations) and manual alignment-based correction.

### Illumina amplicon sequencing

The partial genome consensus sequence generated from initial metagenomic sequence analysis was used to design primers to generate three PCR amplicons (UTR-Amp1 [Forward: GAA TGC TCA CAG AGT CTG C, Reverse: TCG GCG TCT TCT CCA]; UTR-Amp2 [Forward: AAG CGA AGG AGA CAT CC, Reverse: TCG GCG TCT TCT CCA]; UTR-Amp3 [Forward: AAG CGA AGG AGA CAT CC, Reverse: AGA ACC TTG CCC AGC]) covering the unsequenced region of the UTR. PCR amplification of the extracted lung and liver-derived nucleic acid was conducted using the SuperScript™ III One-Step RT-PCR System with Platinum™ Taq DNA Polymerase kit (ThermoFisher) according to manufacturer’s recommendations. The PCR mixture consisted of 2 µL of extracted nucleic acid, 0.3 µM of each primer, and 2 µL of SuperScript™ III RT/Platinum™ Taq Mix in 1 × reaction buffer in a final volume of 50 µL with UltraPure Distilled Water (Sigma-Aldrich). Amplification conditions were denaturation at 94 °C for 2 min followed by 40 PCR cycles with denaturation at 94 °C for 15 s., annealing at 55 °C for 30 s. and extension at 68 °C for 1 min. with a final extension step of 68 °C for 5 min. PCR product was visualized using a QIAxcel instrument (QIAGEN) and prepared for sequencing using the Nextera XT Library Prep Kit (Illumina), and sequenced on the Illumina MiSeq with a V2 flow cell using a 300 cycle kit (Illumina).

### Illumina amplicon sequencing assembly

Amplicon sequencing reads from lung and liver tissue were combined and mapped to the partial genome consensus sequence previously generated from metagenomic sequencing data, using Geneious (v9.1.8)^29^ iterative reference assembly (using default medium–low sensitivity settings and five iterations). The consensus sequence was further analyzed using an alignment-based manual correction method.

### Nanopore amplicon sequencing

Oxford Nanopore long-read sequencing was used for subsequent amplicon sequencing to generate long reads covering the whole unsequenced portion of the UTR. Previously designed primers were used to generate amplicons UTR-Amp1, UTR-Amp2, and UTR-Amp3 using a PCR enzyme designed for amplification of GC-rich targets. Lung-derived nucleic acid was amplified using Invitrogen Platinum SuperFi II (ThermoFisher) according to manufacturer’s recommendations. The PCR mixture consisted of 2 µL of extracted nucleic acid, 0.5 µM of each primer, and 5 uL of 5X SuperFi II Buffer, brought up to a final volume of 20 µL with UltraPure Distilled Water (Sigma-Aldrich). Denaturation was carried out at 98 °C for 30 s. followed by 40 cycles of denaturation at 98 °C for 10 s., annealing at 60 °C for 10 s. and extension at 72 °C for 2 min. with a final extension step of 72 °C for 5 min. The product was visualized using a QIAxcel (QIAGEN) and prepared for sequencing using the Oxford Nanopore Ligation Sequencing Kit (SQK-LSK-109, Oxford Nanopore Technologies) according to manufacturer’s recommendations. Sequencing was conducted on a GridION sequencer (Oxford Nanopore Technologies) with live basecalling enabled (high-accuracy basecalling model) using MinKNOW (v20.06.9).

### Nanopore amplicon assembly

Nanopore reads were trimmed for adapters using Porechop (v.0.2.4)^30^ on default settings. To filter for reads containing the entire region of interest, the trimmed reads were first reference mapped in Geneious (v9.1.8)^29^ (default settings, medium sensitivity) to a 40 nt sequence within the amplicon but flanking the unknown region (ATAAAGGCATAGTCCCAATCCCACCAACGCACAAAAAGAG). The resulting mapped reads were then mapped to a 40 nt sequence flanking the opposite side of the unknown region (GAACGGAGCGAAGCCCGTGGAGTTAAGGGGCAACTCGGGC). The resulting mapped reads containing both known flanking regions were size filtered in Geneious (v9.1.8)^29^ to generate a list of reads with a length similar to the estimated amplicon size (1200–1400 nt for Amp1, 950–1350 nt for Amp2 and 725–1125 nt for Amp3). The resulting filtered reads were aligned using MAFFT^31^ on default settings, and from this alignment a majority consensus sequence was generated in Geneious (v9.1.8)^29^ for each amplicon. The previously unsequenced region of the UTR was extracted from each amplicon, and then they were aligned using MAFFT^31^ on default settings. From this alignment, a single consensus sequence was generated in Geneious (v9.1.8)^29^. This consensus sequence representing the unsequenced region of the UTR was added to the previously generated partial genome sequence to generate a preliminary complete genome sequence for further analysis.

### Final assembly

Previously generated Illumina amplicon sequencing reads were processed using BBMerge^32^, which identified overlapping regions in paired reads, and if present, combined them to generate longer merged reads. Merged reads were mapped to the preliminary complete genome sequence using Geneious (v9.1.8)^29^ iterative reference assembly (using default medium–low sensitivity settings and five iterations). The resulting consensus sequence was modified using an alignment-based manual correction method, resulting in a 2,805 nt complete circular genome. As an additional quality check, sequencing reads were mapped to the final consensus sequence using Geneious (v9.1.8)^29^ reference assembly (default low sensitivity settings).

### Phylogenetic analysis

**Figure 1:**
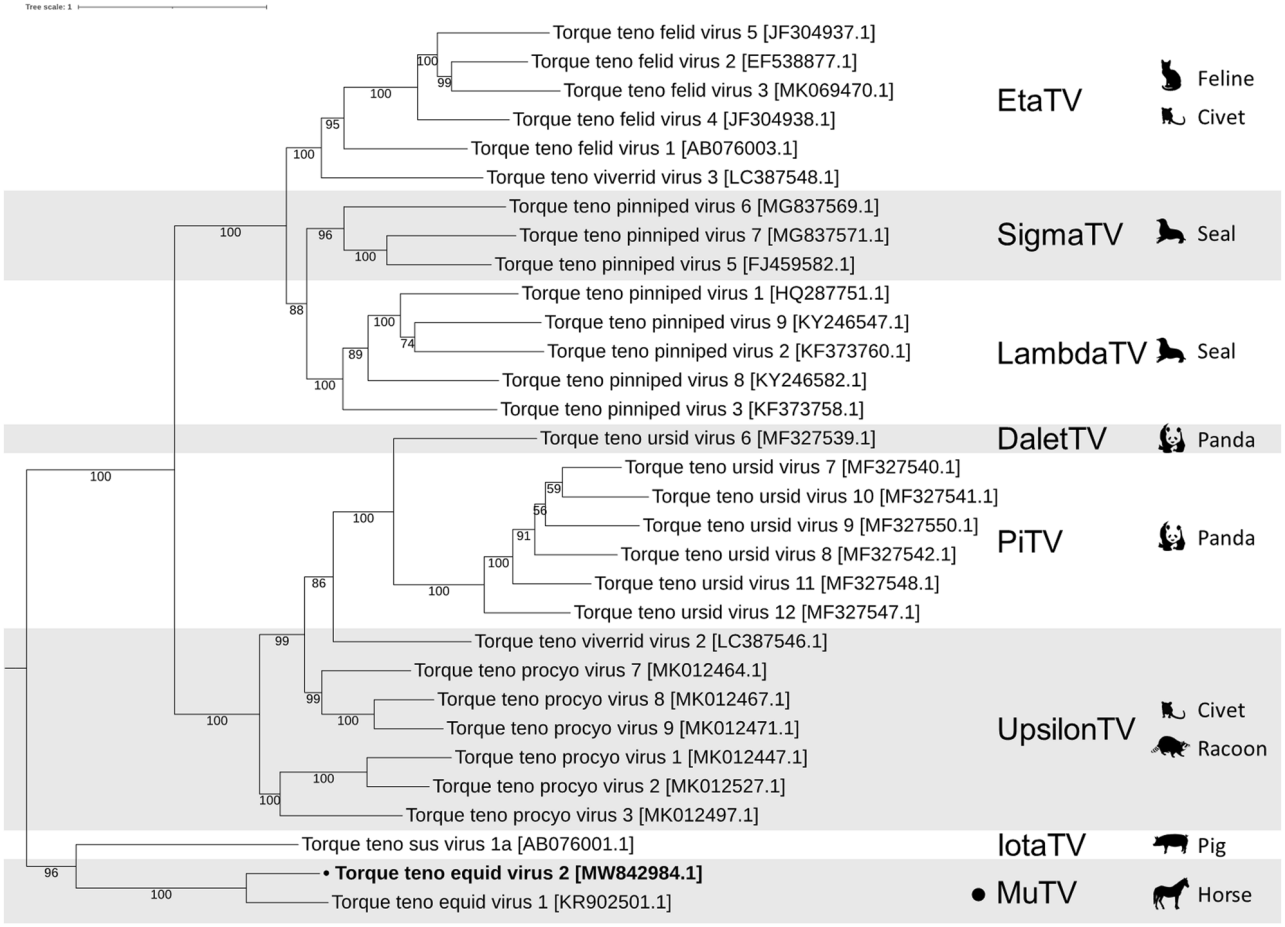
Maximum likelihood phylogenetic trees of representative torque teno viruses. ORF1 amino acid sequences were aligned using MAFFT^31^, trees generated using IQ-TREE^33^ on find best model setting with ModelFinder^34^ (VT + F + R6 model selected) with 1000 ultrafast bootstraps^35^ and visualized using iTOL^36^. Genus names are indicated on the right with “-torquevirus” shortened to “TV” and the associated host species for each genus shown. The novel TTEqV2 and its genus (*Mutorquevirus*) and both indicated with a “black filled circle”. The tree shown here was pruned to include only the clade containing TTEqV2 and its sister clade with the whole unpruned tree available in Supplementary Material Fig. S1.

A maximum-likelihood phylogenetic tree was generated with IQ-TREE^33^ from MAFFT^31^ multiple sequence alignments (MSA) of the novel genome and representative TTV ORF1 amino acid sequences (n = 146). An IQ-Tree phylogenetic tree was produced using the substitution models indicated in Fig. 1, as selected by ModelFinder^34^, with 1000 ultrafast bootstraps^35^ and visualized using Interactive Tree Of Life (iTOL)^36^. The resulting tree was pruned to include only the clade containing TTEqV2 and its sister clade (n = 31) as shown in Fig. 1. The whole unpruned tree is shown in Supplementary Material Fig. S1.

### Nucleotide composition and codon usage analysis

Nucleotide composition and codon usage analysis were performed using CAIcal^37^ (standard genetic code setting) with the same database of representative TTV ORF1 sequences used for phylogenetic analysis (n = 146) with the addition of representative gyrovirus sequences (n = 10). Six TTV sequences were removed from the database and not included in this analysis due to issues that caused errors in CAIcal^37^. Several had degenerate bases (AB025946.2, AB038621.1, JF304938.1, KF764701.1, and KX611132.1), while a single sequence (DQ187006.1) had a total number of ORF1 nucleotides not divisible by 3. Relative synonymous codon usage (RSCU) values for host organisms were obtained from a previous study^38^. A spreadsheet containing the nucleotide composition results from CAIcal for all sequences as well as the RSCU values for selected anelloviruses and associated hosts is available in Supplementary Material Table S2.

## Results

**Figure 2:**
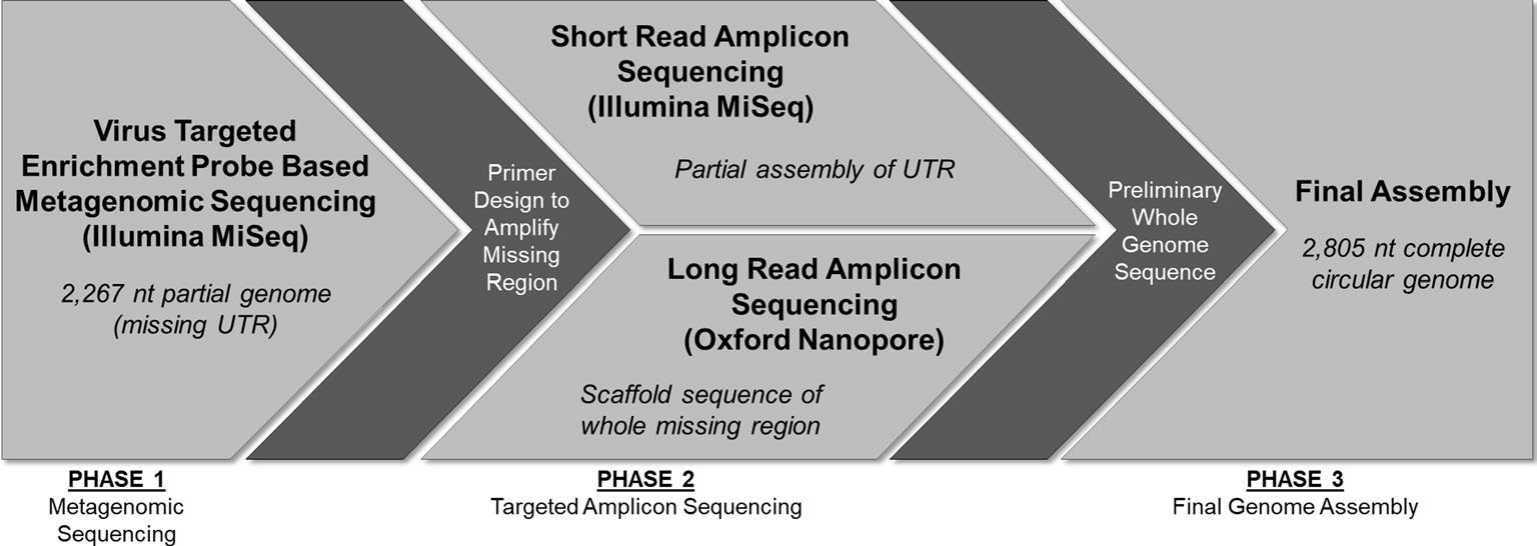
Schematic of workflow used for generation of novel TTEqV2 complete circular genome sequence.

The workflow used for sequencing and assembly of the complete novel TTV genome utilized a combination of capture probe enrichment, Illumina short-read amplicon sequencing, and Nanopore long-read amplicon sequencing (Fig. 2). Following initial metagenomic sequencing, 2103 and 1892 nt TTV contigs were observed in the lung and liver tissue derived samples, respectively. Nucleic acid derived from other tissues was not incorporated into further analysis because the kidney and spleen samples did not generate TTV contigs while the brain tissue did not generate useful reads, likely due to nucleic acid degradation resulting from storage in formalin. The lung and liver tissue derived contigs were 100% identical in overlapping regions, and were thus combined to generate a single 2267 nt consensus sequence.

**Figure 3:**
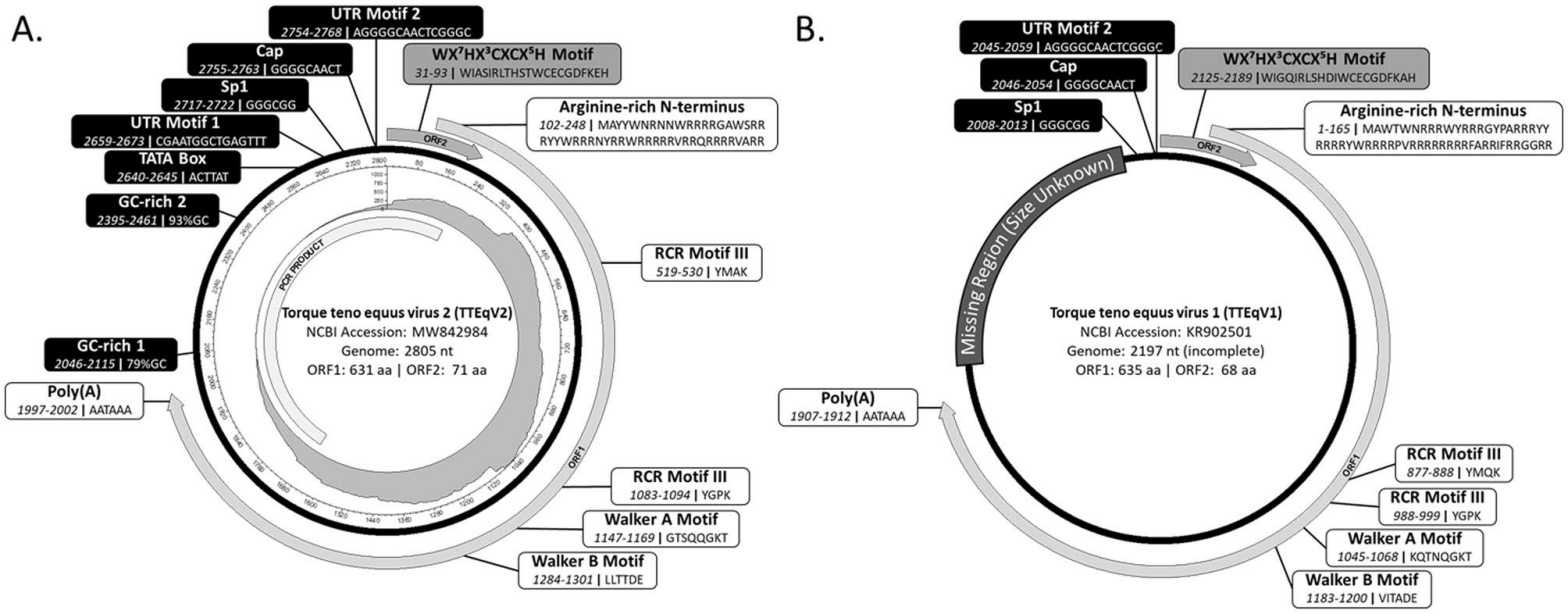
Genomic structure of (**A**) Torque teno equus virus 2 (TTEqV2) and (**B**) Torque teno equus virus 1 (TTEqV1). Inside the annotated circular genome for TTEqV2 is a plot showing the coverage at each nucleotide position (generated using circlize v0.4.15^58^). The region sequenced via subsequent targeted amplicon sequencing is indicated by a bar within the coverage plot. For both viruses, genomic features of interest are labelled with the feature name followed by the position and sequence (or GC content for GC-rich regions in TTEqV2) below.

Alignment to existing TTV sequences suggested that a portion of the UTR was missing from the 2267 nt consensus sequence. This missing region was determined using amplicon sequencing with primers targeting the regions flanking the missing region, determined by initial metagenomics sequencing. Initial Illumina short-read amplicon sequencing did not generate the complete missing region, likely due to difficulty assembling several GC-rich homopolymeric regions and lack of a suitable reference genome for read mapping. Subsequent Nanopore long-read sequencing was used to generate a scaffold which was used in combination with existing Illumina data to generate a sequence for the entire amplified region. Consensus sequences from metagenomic and amplicon sequencing were combined to generate a 2805 nt final consensus sequence with 50% GC content. Both metagenomic and amplicon sequencing reads mapped across the entire final consensus sequence, including the linearization point of the circular genome, indicating this sequence represents the complete circular TTEqV2 genome. The structure of the complete circular TTEqV2 genome is shown in Fig. 3.

The TTEqV2 genome shares several common characteristics with previously reported TTVs. ORF1 is the longest ORF and encodes a 631 aa protein with an arginine-rich N-terminus (MAYYWN**R**NNW**RRRR**GAWS**RRR**YYW**RRR**NY**RR**W**RRRRR**V**RR**Q**RRRR**VA**RR**), conserved amino acid motifs and a downstream polyadenylation signal (Fig. 3). While ORF1 encodes a capsid protein, it contains four rolling circle replication (RCR) or helicase activity associated amino acid motifs (two RCR IIIs [YGPK and YLTK], one Walker-A [GTSQQGKT] and one Walker-B [LLTTDE]) that have also been found in other circular ssDNA virus genomes^9^. There is also an ORF2 encoding a 71 aa putative protein, in the same orientation and overlapping with the N-terminal end of ORF1, that contains a highly conserved WX_7_HX_3_CXCX_5_H motif. Based on analysis using SnapGene Viewer v5.0.7 (snapgene.com), the novel TTV genome contains six additional ORFs, encoding hypothetical proteins > 50 aa, ranging in size from 59 to 137 aa. HMMer3 hmmsearch^39^ against the Pfam HMM DB (v33.1)^40^ (performed February 22, 2023) showed that ORF1 and 2 matched ORF1 and 2 from other TTVs, respectively, while the six additional ORFs had no matches. Blastp^28^ analysis on default settings using the nr database showed consistent results, with matches to TTV ORFs for ORF1 and 2 but no matches for any of the six additional ORFs (performed February 22, 2023).

**Figure 4:**
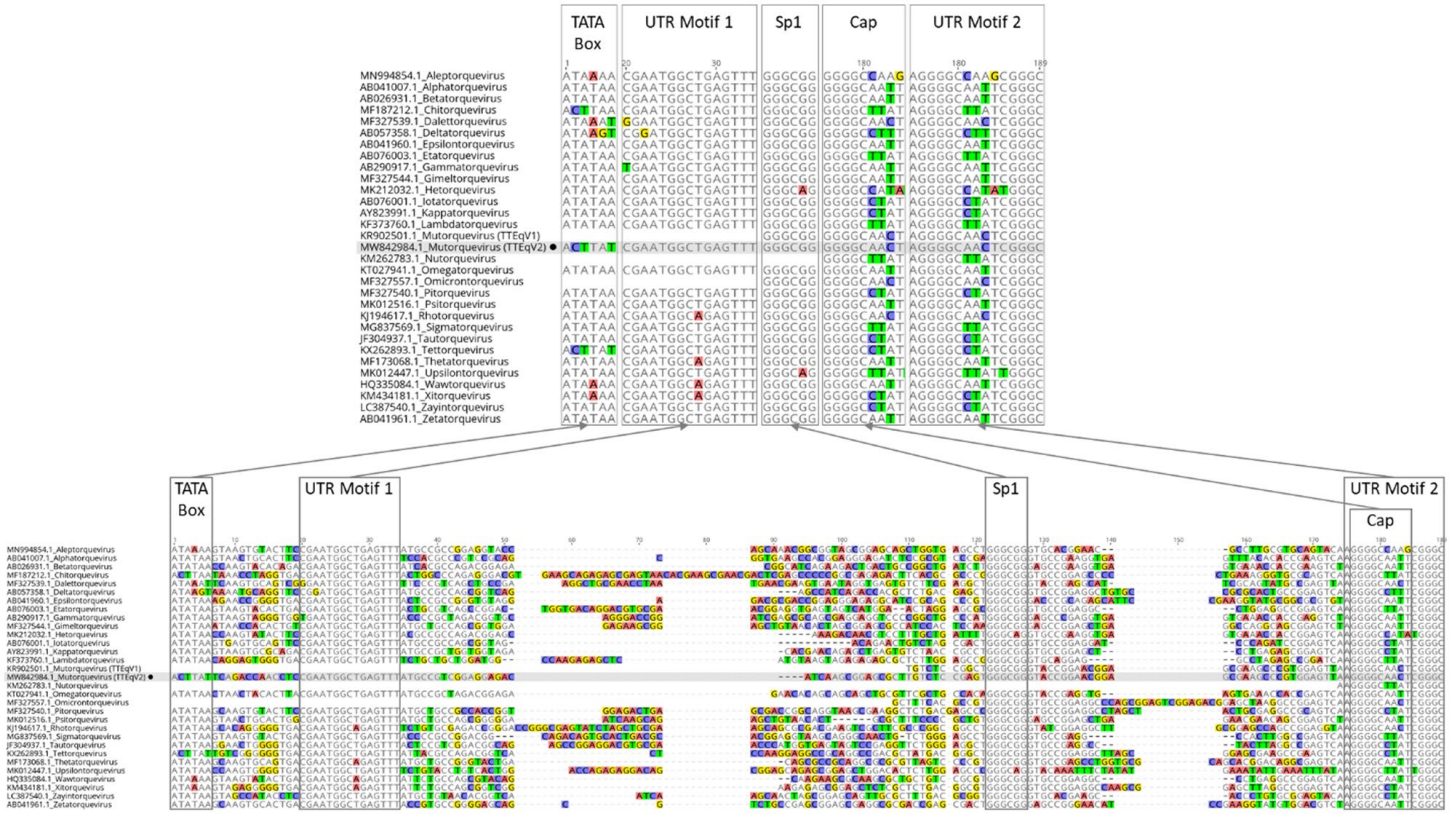
Nucleotide alignment of conserved region of the UTR from TTV genomes representing every currently recognized genus. Putative transcription factors and conserved motifs are labelled and outlined with boxes. Highlighted bases indicate those that differ from the majority consensus sequence. Each sequence is labelled with the NCBI accession number and genus. The novel TTEqV2 sequence is indicated with a black filled circle and shaded in gray for emphasis.

Like other TTVs, the novel genome contains a UTR with two 15 nt conserved motifs, putative transcription factors (TATA box, Sp1 site, Cap site, and polyadenylation signal), and GC-rich region (Figs. 3 and 4). The two 15 nucleotide conserved sequence motifs within the UTR, hereinafter referred to as UTR motif 1 and 2, are similar to those previously described in other TTV genomes^5–7^ (Fig. 4). When compared to these previously reported UTR motifs, TTEqV2’s UTR motif 1 has 100% identity (CGAATGGCTGAGTTT) while motif 2 has a single nucleotide substitution (AGGGGCAA[T>C]TCGGGC). The TATA box, identified based on its position relative to UTR motif 1 (13 bp upstream), appears to be conserved in TTVs as shown in the alignment in Fig. 4. Interestingly, TTEqV2 contains an atypical putative TATA box (ACTTAT) which differs from the canonical TATA box seen in most TTVs (ATATAA).

The most closely related publically available genome to TTEqV2 is TTEqV1 (KR902501) with an ORF1 pairwise nucleotide identity of 59.7% and amino acid identity of 52.5%. A phylogenetic tree built using representative TTV ORF1 sequences demonstrates that the novel TTEqV2 clusters with TTEqV1 (Fig. 1). Pairwise alignment of the two TTV equine sequences with MAFFT^31^ using the default settings shows that TTEqV1 is missing several genomic features conserved in TTVs, including UTR motif 1 and a GC-rich region (Figs. 3 and 4).

Nucleotide composition analysis of ORF1 determined that in both TTEqV2 and TTEqV1, adenine was the most abundant nucleotide at 36.5% and 35.4%, respectively. When similar analysis was performed on a database of representative anellovirus sequences a similar trend was seen, with 138 of 150 (92%) total sequences having adenine as the most abundant nucleotide, an average abundance of 35.5% and a minimum abundance of 24.4%. When gyroviruses were removed, the number of sequences with adenine as the most abundant changed to 134 out of 140 (95.7%), with an average abundance of 35.9%. Interestingly, the six TTV sequences where adenine was not the most abundant all had cytosine as the most abundant nucleotide and came from either a primate (KP296853.1 [27.3%A], KP296854.1 [29.4%A], KP296856.1 [30.5%A] and AB041961.1 [24.4%A]) or feline host (KX262893.1 [28.1%A] and AB076003.1 [26.3%A]) (Supplemental Material S1).

**Figure 5:**
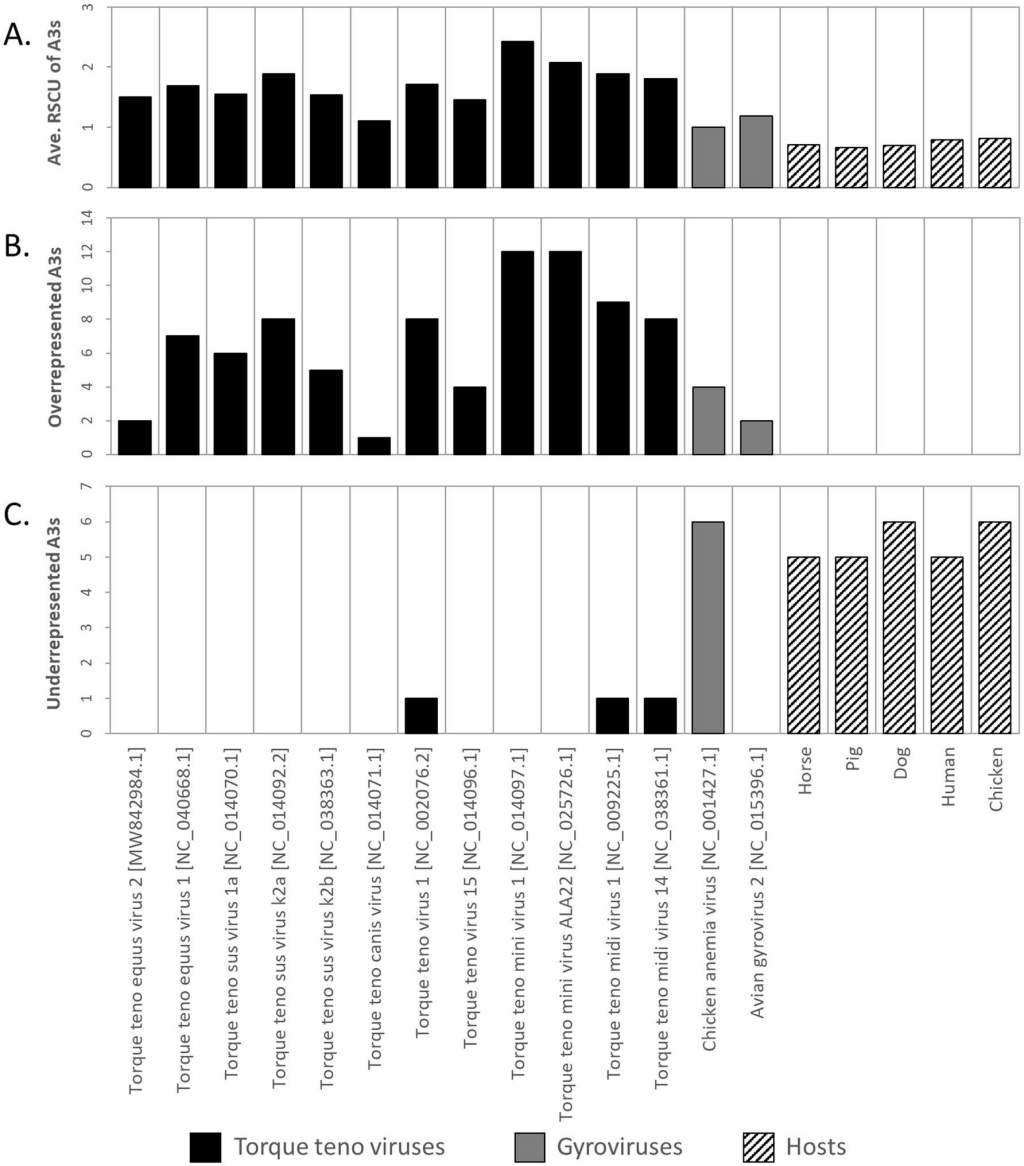
Codon usage analysis of adenine ended codons (A3s) in TTVs, gyroviruses and associated host species. (**A**) Average relative synonymous codon usage (RSCU) of A3s. (**B**) Number of overrepresented A3s (those with RSCU values > 1.6). (**C**) Number of underrepresented A3s (those with RSCU values < 0.6). A spreadsheet containing the RSCU values for the anelloviruses and associated hosts shown here is available in Supplementary Material Table S2. All RSCU values for host organisms were obtained from a previous study^38^.

Codon usage analysis of anellovirus ORF1 sequences from genomes representing eight genera, selected based on the availability of codon usage data for associated host species, revealed a bias toward adenine ending (A3) codons in the anelloviruses (Fig. 5). Here, relative synonymous codon usage (RSCU), a measure of the frequency of a specific synonymous codon versus the expected frequency without bias, was used to compare codon usage patterns among anellovirus and host genomes. All anellovirus ORF1 sequences analyzed had at least one overrepresented A3 codon (RSCU > 1.6). Similar analysis performed on the associated host species (horse, swine, canine, human, and chicken)^38^ found that none of them had a single overrepresented A3 codon. Analysis of underrepresented codons (RSCU < 0.6) determined that of the twelve total anellovirus ORF1 sequences analyzed, three of the human TTVs had a single underrepresented A3 codon, while none of the six non-human TTVs had any underrepresented A3 codons. It is worth noting that the underrepresented A3 codon for all three human TTVs was CGA. In all cases AGA, another A3 codon which codes for the same amino acid (arginine), was highly overrepresented (all RSCU > 3). The number of under-represented A3 codons ranged from 0 to 6 in the gyroviruses and from 5 to 6 in the analyzed host species. Average RSCU values for A3 codons were greater than or equal to 1 for all anelloviruses and less than 1 for all host species analyzed.

## Discussion

A 2,197 nucleotide TTEqV1 genome identified in the metagenomic analysis of plasma from a horse was previously the only sequence within the genus *Mutorquevirus*^41^. Our analysis suggests that the reported TTEqV1 genome sequence is incomplete and missing a portion of the UTR region including one of the two conserved 15 nt sequences and a GC-rich tract, both highly conserved features in the UTR of TTVs. An ORF with homology to the ORF2 identified in TTEqV2 (including the highly conserved WX_7_HX_3_CXCX_5_H motif) was also observed in our analysis of TTEqV1, but is not annotated on the NCBI entry.

The novel TTEqV2 genome contains several genomic features with varying levels of similarity to those previously described in other TTV genomes. ORF1 and ORF2 have similar size, position, and amino acid motifs to other publicly available TTV sequences. TTEqV2 and TTEqV1 have similar amino acid motifs within ORF1; however, some differ in position and/or sequence. The ORF1 of both genomes contain two RCR motif IIIs, one of which is in a similar position and has an identical amino acid sequence (YGPK), while the other has both a different position and sequence (YMQK in TTEqV1, YMAK in TTEqV2). The Walker-A and B motifs are in a similar position in both genomes but differ in amino acid sequence (KQTNQGKT for Walker-A and VITADE for Walker-B in TTEqV1, GTSQQGKT for Walker-A and LLTTDE for Walker-B in TTEqV2).

Two GC-rich regions, characteristic of TTV genomes, are located within the UTR of TTEqV2. The first is 70 nt with 78.6% GC, while the second is 67 nt with 92.5% GC. These GC-rich regions, which contain long homopolymeric stretches, were likely the reason initial analysis with only metagenomic data failed to generate a complete genome sequence. Assembly of the final genome required a combination of metagenomic, short, and long-read amplicon sequencing. Similarly, when the first human TTVs were sequenced, it was thought to be a linear genome due to difficulty amplifying and sequencing GC-rich regions^42^.

Transcription regulatory sites identified in TTEqV2, including the Sp1 site, cap site, and polyadenylation signal, are similar to those characterized in other TTV genomes. The Sp1 site and polyadenylation signal exactly match those described in previously characterized TTV genomes, while the Cap site has a single nucleotide difference which is also seen in TTEqV1 (GGGGCAA[T>C]T)^4,5^. The TATA-box, which is well conserved in most TTV genomes, appears to be either heavily modified or missing from the expected region of TTEqV2. Generally, TTV genomes have a TATA-box that is 13 nt upstream of UTR motif 1 and conforms to the canonical consensus sequence (ATATAA) with slight variations in some cases. The putative atypical TATA-box in TTEqV2 (ACTTAT), determined based on location relative to the conserved motif, has three nucleotide differences compared to the canonical sequence. The  incomplete TTEqV1 genome does not include UTR motif 1 or the upstream region containing the TATA-box, so the sequence of this region in the other available *Mutorqevirus* genomes is unknown. However, an identical atypical putative TATA-box is seen in the representative *Tettorquevirus* genome (KX262893.1), and one with a single base difference (ACTTA**A**) is seen in the representative *Chitorquevirus* genome (MF187212.1). Both of these representative genomes are the only publically available species within their genus, so whether this atypical putative TATA box is conserved in other sequences of the genus is unknown. Interestingly, neither of these sequences cluster with TTEqV2 based on the alignment of ORF1 and come from different host species (*Tettorquevirus* from feline and *Chitorquevirus* from lemur).

Nucleotide composition analysis revealed that anellovirus ORF1 sequences tend to be adenine rich, with A3 codons favoured in the sequences analyzed. Previous studies made similar observations in anelloviruses^43^, swine TTV^44^ and equine influenza virus sequences^38^. Interestingly, the opposite trend was observed in the associated host species (horse, pig, dog, human and chicken) for all anellovirus genera analyzed, where A3 codons were underrepresented. A previous study suggested that if codon usage bias in a virus is too similar to that of the host, host translation may be impeded, leading to a greater chance of the virus generating a symptomatic response in the host^45^. The significance of the observation that the TTEqV2 genome has dissimilar codon usage compared to its equine host remains to be determined.

Although TTV has been proposed to be related to many diseases, there are only a few reports supporting the disease-inducing potential of TTV^1^. Human TTVs have been proposed to play a role in the pathogenesis of certain diseases, such as hepatitis^46^, hematological disorders^47^, respiratory diseases^48^, rheumatic autoimmune disease^49^. A recent viral metagenomic study identified a novel betatorquevirus species prevalent in pediatric encephalitis/meningoencephalitis cases, but absent in healthy cohorts^5^.

Torque teno sus viruses (TTSuVs) have been found at a particularly high frequency in healthy swine^50,51^. While considered non-pathogenic on their own, there is increasing evidence that TTSuVs may influence the development or outcome of some diseases^52^. For example, co-infection with porcine circovirus type 2 (PCV2) and the associated porcine circovirus diseases deserve special attention^53^. TTSuVs have also been partially attributed to inducing porcine reproductive and respiratory syndrome, porcine dermatitis and nephropathy syndrome, and hepatitis^54,55^. TTSuV2 viremia may be associated with the level of immunocompetence of the animals^52^. A study with pigs infected with hepatitis E virus has shown a correlation between TTSuV and the increased risk of developing severe hepatitis in animals co-infected with PCV2^56^. A high prevalence of TTSuV1, but not TTSuV2, in pigs suffering from porcine respiratory disease complex has been shown^57^. Such viruses would likely be considered components of the host microbiota and unable to cause disease directly, but instead available to be engaged in physiological processes and modulate the organism's response to other pathogens^1^. The relationship between TTV, disease and host immune response is not well understood and therefore the connection between TTEqV2 and the disease observed in the horse, if any even exists, remains to be determined.

In conclusion, this study describes the discovery of a novel anellovirus species which represents the first complete genome within the genus *Mutorquevirus*. Comparative genomic analysis showed that TTEqV2 shares many conserved features with previously reported TTVs and it has been recognized as a novel species by the ICTV^13^. This, along with previous studies using similar methods^15,16,18,19^ demonstrates the power of HTS for characterization of unexpected and/or novel viruses in a variety of hosts and sample types.

## Supplementary Information

Supplementary Figure S1.

Supplementary Table S2.

## Data Availability

The complete genome is available on NCBI under accession MW842984.
