# Supplementary material for: Discovery and comparative genomic analysis of a novel equine anellovirus, representing the first complete Mutorquevirus genome
Source: Sci Rep. 2023 Mar 6;13:3703. doi: 10.1038/s41598-023-30875-7 (PMC9988894; doi:10.1038/s41598-023-30875-7)

Supplemental material S1

**Maximum likelihood phylogenetic tree of representative torque teno viruses.** ORF1 amino acid sequences were aligned using MAFFT31, trees generated using IQ-TREE33 on find best model setting with ModelFinder34 (VT+F+R6 model selected) with 1,000 ultrafast bootstraps35 and visualized using iTOL36. The novel TTEqV2 indicated with a “●”.


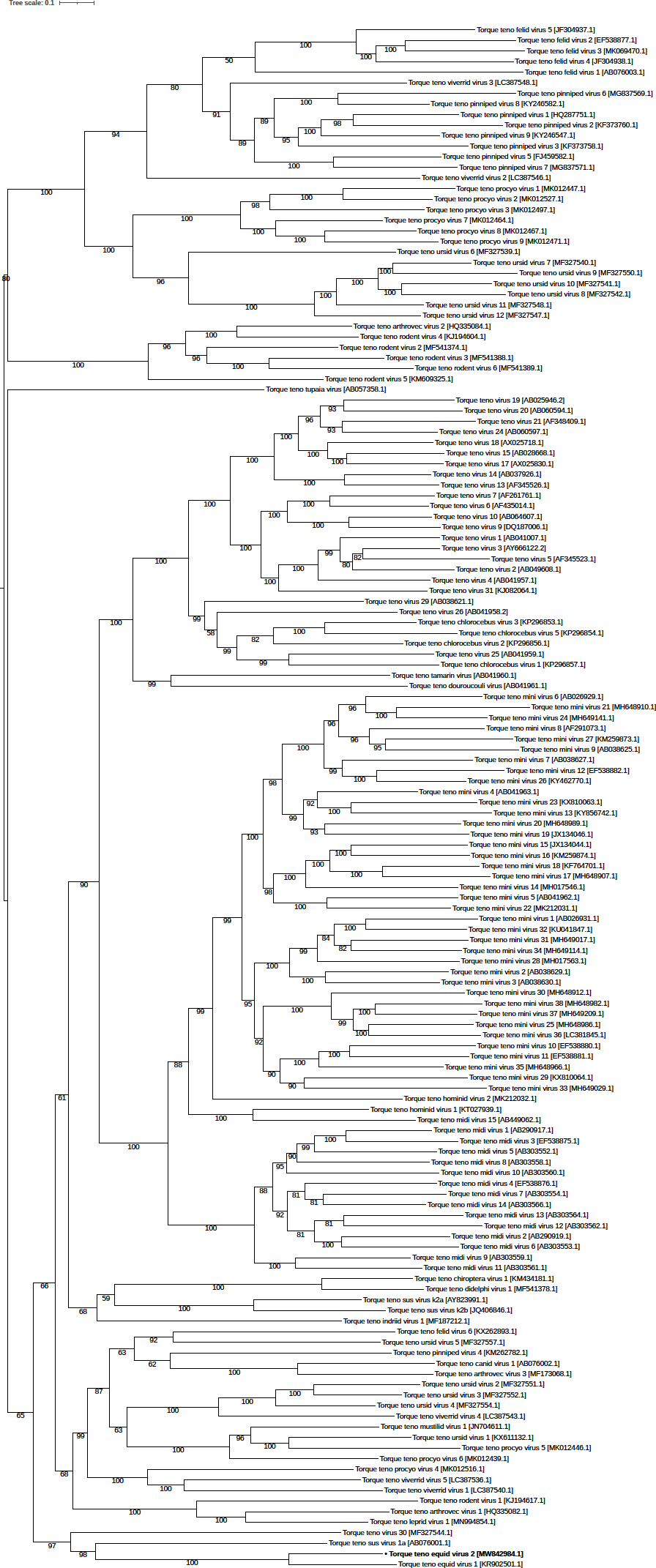

Supplement: Supplementary file 1 — Supplementary Figure S1. [file 41598_2023_30875_MOESM1_ESM.docx]
